# Editorial: New generation bacterial vaccines based on immune correlates of protection

**DOI:** 10.3389/fimmu.2026.1909196

**Published:** 2026-09-25

**Authors:** Mehak Z. Khan, Aditya Kumar Sharma, Kirtimaan Syal

**Affiliations:** 1 The Ragon Institute of Mass General Brigham, MIT, and Harvard, Cambridge, MA, United States; 2 Department of Pathology, College of Medicine, University of Illinois at Chicago, Chicago, IL, United States; 3 Genetics and Molecular Microbiology Laboratory, Department of Biological Sciences, Birla Institute of Technology & Science, Pilani, Hyderabad, Telangana, India

**Keywords:** adaptive immunity, adjuvant, immune correlate of protection, multiepitope based vaccine, peptide based vaccine, pneumonia

Vaccines remain the most effective defense in the fight against the evolving bacterial pathogens. The emergence of multidrug resistance, the capacity to evade human immune response, and persistence as chronic infections have made bacterial diseases increasingly difficult to treat. The threat of antimicrobial-resistant pathogens is has been exacerbated by the overuse/misuse of antibiotics, which has accelerated the natural resistance process, leading to a projected loss of 1.1% of the annual global gross domestic product by 2030 and incurring an approximate global economic burden of US$17.5 trillion between 2020 and 2050 (1, 2). The traditional development of antibacterial vaccines has employed several approaches. First-generation vaccines rely on the empirical attenuation or inactivation of bacteria to produce live attenuated and whole-cell inactivated vaccines, respectively. Second-generation vaccines are subunit vaccines based on the purification of specific antigens, such as polysaccharides and toxins, which may be conjugated to carrier proteins when necessary. The latest generation of antibacterial vaccines is rationally designed based on an understanding of both bacterial virulence factors and the correlates of protective immunity. Such correlates include functional antibody activity, mucosal immunity, T-cell responses, innate immune activation, cytokine signatures, immune memory, and protection-linked readouts such as pathogen clearance, reduced colonization, diminished tissue damage, and prevention of dissemination.

This Research Topic, “New Generation Bacterial Vaccines Based on Immune Correlates of Protection,” includes original research and review articles focused on the use of computational vaccinology, mRNA platforms, optimizing adjuvants and live-attenuated vaccine engineering, which could enable protective immunity against bacterial pathogens (**Figure 1**).

**Figure 1:**
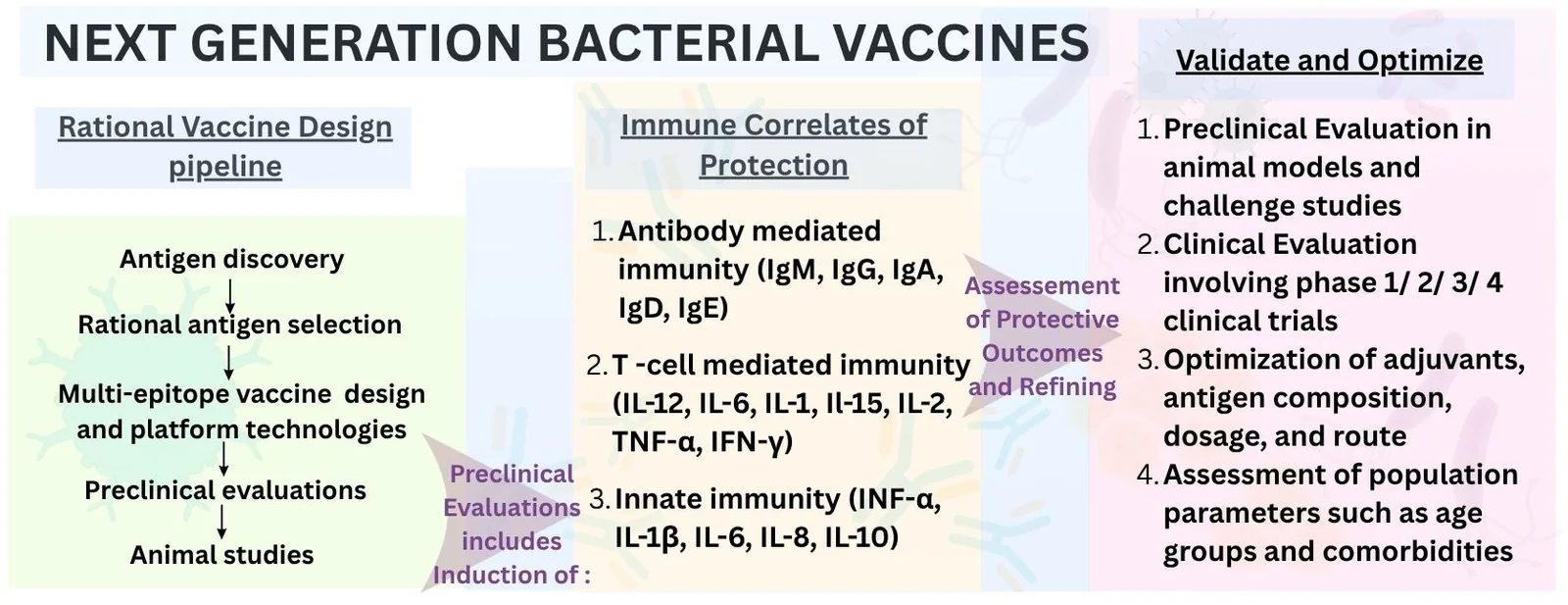
Schematic presentation of the rational design and validation of new generation bacterial vaccines. Vaccine efficacy is measured by key well-known and novel immune correlates of protection. Vaccine optimization is guided by pre-clinical and clinical studies (Designed using Canva, www.canva.com).

A central theme of this collection includes the use of multi-epitope and reverse vaccinology approaches, where multiple epitopes from different surface antigens could be tailored with linkers for effective immune response. Devarakonda et al. showed experimental validation and assessment of a multiepitope vaccine construct against *Streptococcus pneumoniae*. They evaluated the structure of the multiepitope vaccine construct by circular dichroism and further observed it to be non-hemolytic (3). The vaccine induced antigen-specific antibody responses and cytokines including GM-CSF and TNF-α, in BALB/c mice. This study serves as an important example, where in silico antigen predictions have been subjected to experimental validation, particularly relevant in the context of diverse pneumococcal serotypes and limited protection of available polysaccharide-based vaccines.

Dai et al. further carried out a meta-analysis based on a systematic review of the efficacy and safety of pneumococcal conjugate vaccines (PCV) in children. They reviewed 11 studies, which included 147,274 participants, where PCV reduced the incidence of pneumonia plausibly due to the increased IgG responses against serotypes, including 6B, 9V, 14, 18C, 19F, and 23F. However, the authors also noted variability in serotype-specific antibody responses, and limited reporting of side-effects. The paper highlights the need to optimize vaccine formulations and strategies against emerging non-vaccine serotypes.

Similarly, Chundru and Ghanem also used an immunoinformatics approach and designed a multiepitope subunit vaccine against *Salmonella enterica* serovar Infantis, a multidrug-resistant zoonotic pathogen associated with poultry and foodborne disease. Their analysis identified CsgA as a conserved and immunogenic antigen predicted to interact with chicken TLR2/TLR5 in addition to both MHC-I, and MHC-II. Importantly, this study requires experimental validation, though it was predicted to offer protection against serovar-specific bacterial threats.

Lu et al. developed an mRNA-based LNP vaccine encoding the *Treponema pallidum* antigen TP0954 and studied its immunogenicity in mice and rabbits. The vaccine elicited both humoral and cellular immune responses. TP0954 mRNA showed attenuation of lesion development and reduced treponemal burdens at distant organs, making it a viable alternative vaccine to a recombinant protein-based vaccine.

Yuan et al. investigated the role of the BtpB type IV secretion system effector of Brucella in evading the host immune system. btpB deletion resulted in reduced intracellular bacterial survival while increasing immunogenicity, including robust humoral and cellular immune responses, enhancing Th1 activity, CD8+ T-cell responses, and NK-cell activation. Ming et al. demonstrated that adjuvant-driven epitope hierarchy shaped the hierarchy of FimA immunodominant B-cell epitopes and directly influences vaccine-induced protection against hypervirulent *Klebsiella pneumoniae.* The authors investigated novel protective FimA epitopes and highlighted AddaS03 as the most effective adjuvant for protection against *K pneumoniae*.

Shome et al. assessed immunogenicity and efficacy of *Brucella abortus* S19 vaccination in cattle and buffalo calves. Their results demonstrated that S19 vaccination at 4 × 10^9^ CFU/dose was safe and elicited a humoral response in addition to a robust IFN-γ response. Interestingly, cattle were reported to show better adaptive immune response kinetics than buffaloes, highlighting the need for evaluation of vaccines beyond laboratory animal models.

Caradonna et al. tested the altSonflex1-2–3 Shigella GMMA-based vaccine in mice, rats, and rabbits. The study directly compared O-antigen-specific IgG responses, dose-dependent responses, the role of a booster, and antibody response variability across species. The authors also reported species- and antigen-specific differences. This work suggested that the appropriate selection of animal models and dosage optimization are crucial factors for immune-correlate-guided development.

Chakraborty et al. characterized *Clostridioides difficile* lipoproteins LP1 and LP2 and proposed their role as potential vaccine candidates. Both proteins were predicted to contain CTL, HTL, and B-cell epitopes and were tested in a mouse model. Immunized mice showed serum IgG and fecal IgA responses in challenge experiments. Anti-LP1 and anti-LP2 sera also inhibited bacterial adhesion to gut epithelial cells, thereby providing protection against colonization.

In addition to individual vaccine candidates, several reviews covered platform technologies. Zhang et al. reviewed bacterial outer membrane vesicles (OMV) as nanoplatforms for vaccine studies. OMVs, due to their properties such as natural antigen display and membrane biocompatibility, could also enable vaccine delivery. The review elaborates key translational challenges, including safety and scaling for clinical use.

Xia et al. described the engineering of cell membrane vesicles, including bacterial OMVs and mammalian exosomes, into nanodecoys and antigen delivery systems. Importantly, Xia et al. discussed the role of membrane vesicles in pathogen targeting and host immune activation.

Finally, the review by Al-Azzani et al. summarized host-directed antibacterial approaches, including monoclonal antibodies, CRISPR-based antimicrobials, and aptamers. Together, the articles in this Research Topic broadly cover a rapidly evolving field of bacterial vaccine development. Although most studies presented in this Research Topic are preclinical and require validation in human cohorts or animal models, the approach holds promise for future use. Computationally designed vaccines require systematic experimental testing, whereas mRNA platforms require further optimization for antigen expression. Moreover, as discussed, dosage dependence, routes of administration, and species-specific immune response variability can also affect disease outcomes.

In summary, this Research Topic covers the design of bacterial vaccines, including multiepitope vaccine constructs, their experimental validation, and diverse correlates of immunogenicity and efficacy.

## References

[1] Poudel AN Zhu S Cooper N Little P Tarrant C Hickman M . The economic burden of antibiotic resistance: a systematic review and meta-analysis. PLoS One. (2023) 18:e0285170. doi: 10.1371/journal.pone.0285170 37155660 PMC10166566

[2] Syal K Chatterji D . Vitamin C: a natural inhibitor of cell wall functions and stress response in mycobacteria. In: Chattopadhyay K Basu SC , editors. Biochemical and Biophysical Roles of Cell Surface Molecules. Singapore: Springer Singapore (2018). p. 321–32. doi: 10.1007/978-981-13-3065-0_22 30637707

[3] Devarakonda Y Reddy M Neethu RS Chandran A Syal K . Multi epitope vaccine candidate design against Streptococcus pneumonia. J Biomol Struct Dyn. (2023) 41:12654–67. doi: 10.1080/07391102.2023.2167123 36636838

